# Supplementary material for: Triple-Negative Breast Cancer EVs Modulate Growth and Migration of Normal Epithelial Lung Cells
Source: Int J Mol Sci. 2024 May 28;25(11):5864. doi: 10.3390/ijms25115864 (PMC11172765; doi:10.3390/ijms25115864)
Supplement: Supplementary file 1 [file ijms-25-05864-s001.zip › ijms-3003862-supplementary.pdf]

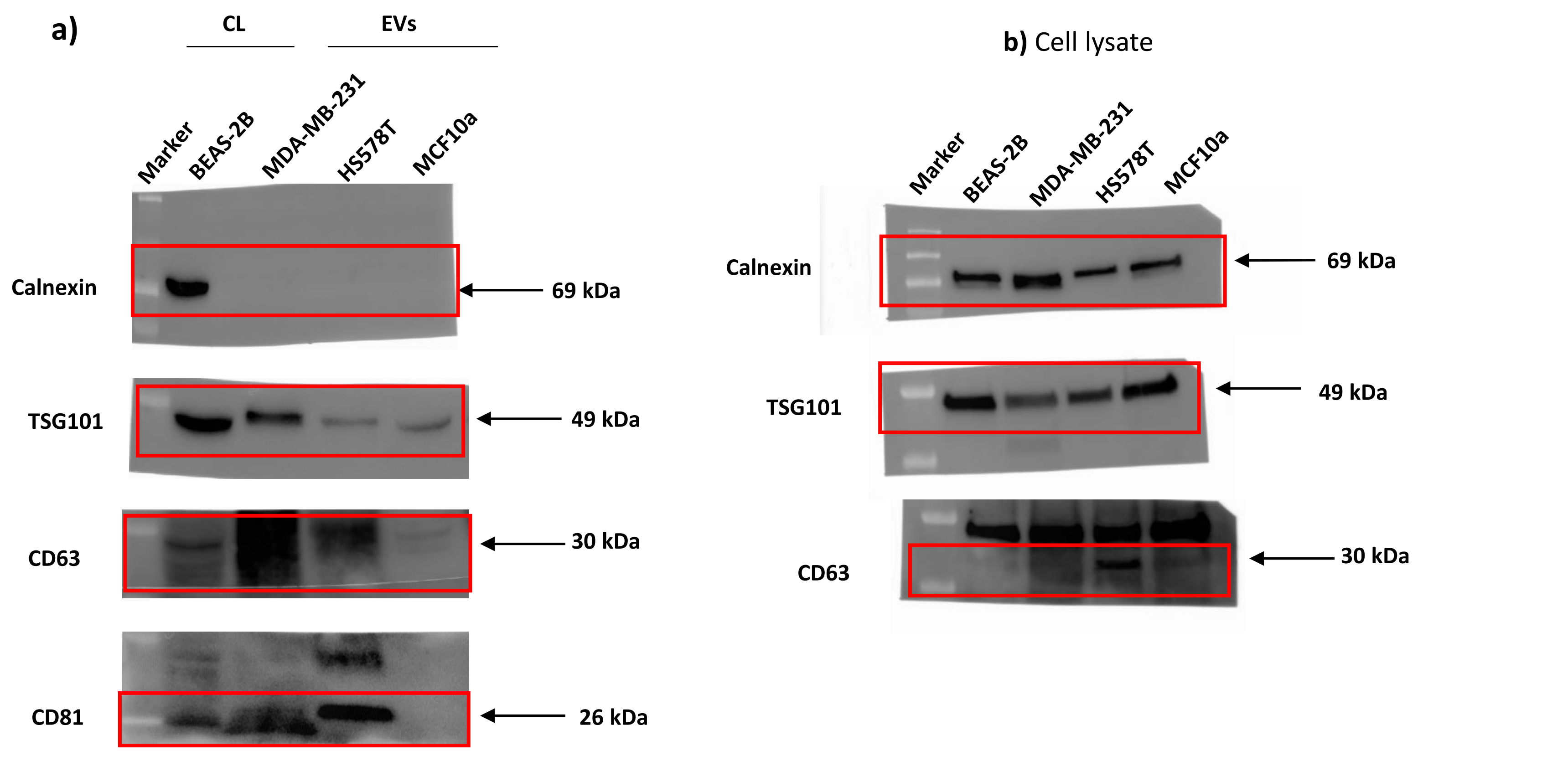

Figure S1. Uncropped WB images reported in Figure 2. Red boxes report the cropped WB images of a) EVs and b) cell lysate from which EVs were isolated

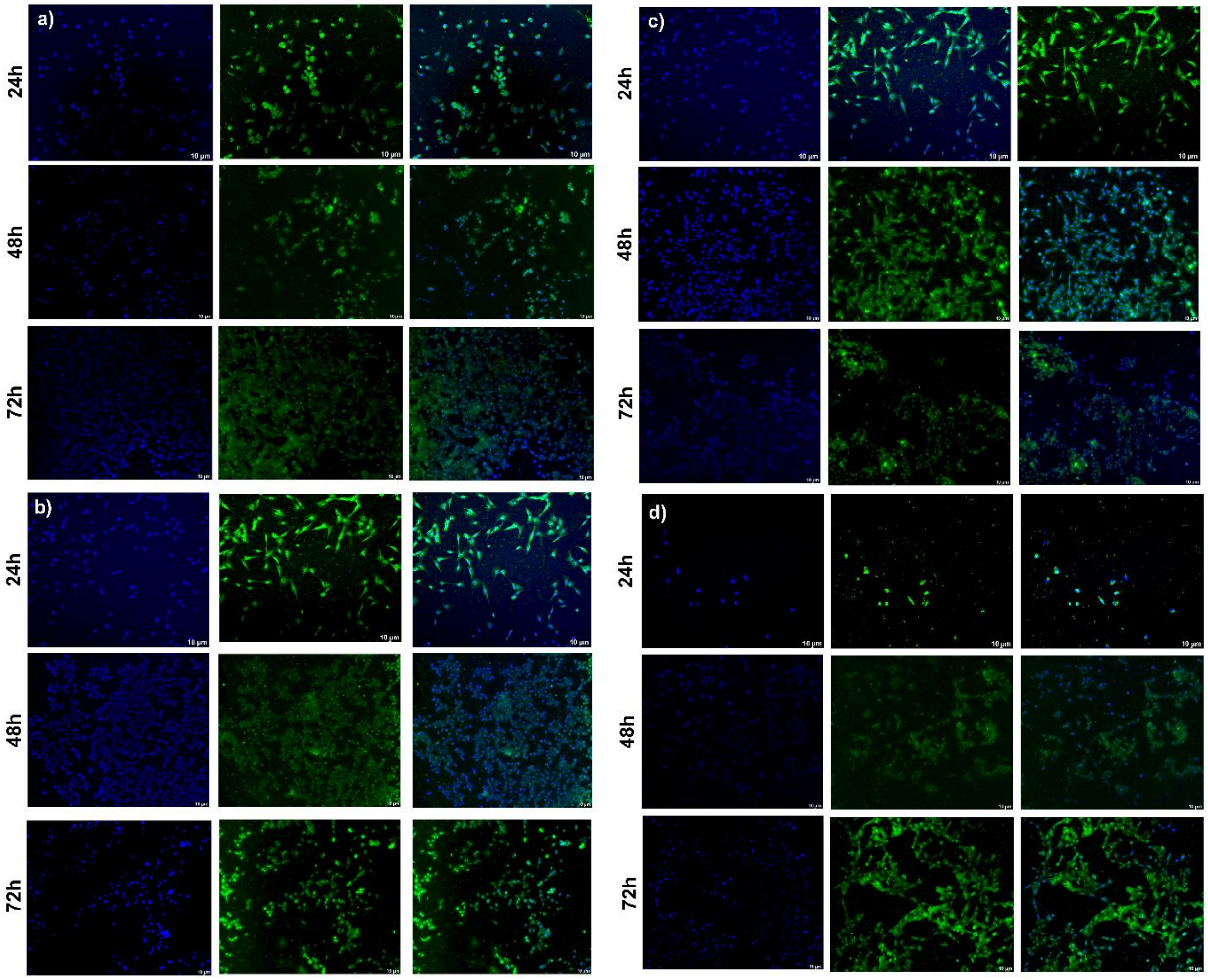

Figure S2: Migration Assay reported in Figure 5. Representative confocal images of BEAS-2B migrated through the Transwell chamber after incubation for 24, 48 and 72h with EVs separated from a) MDA-MB-231, b) HS578T, c) MCF10a cell lines. Cell nuclei were stained with DAPI and Transwell chamber were colored with crystal violet. Scale bar: 10 µm.

a) MDA-MB-231 EVs

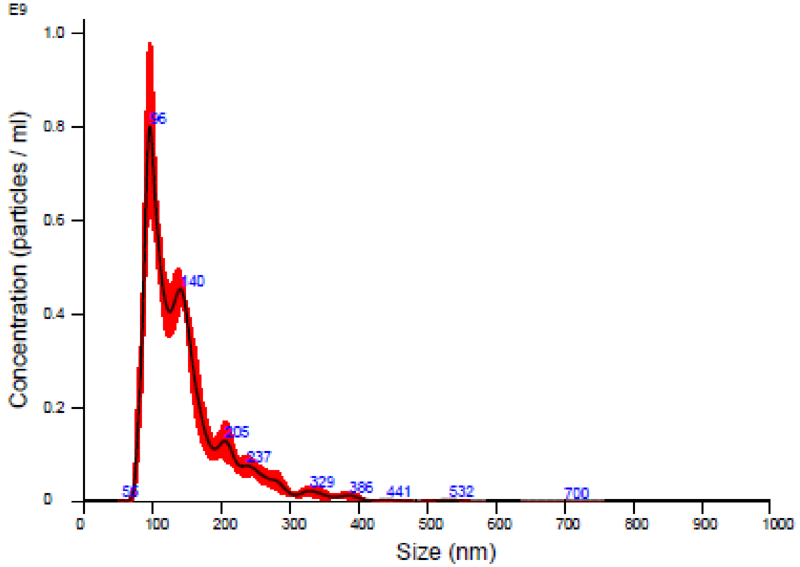

b) HS578T EVs

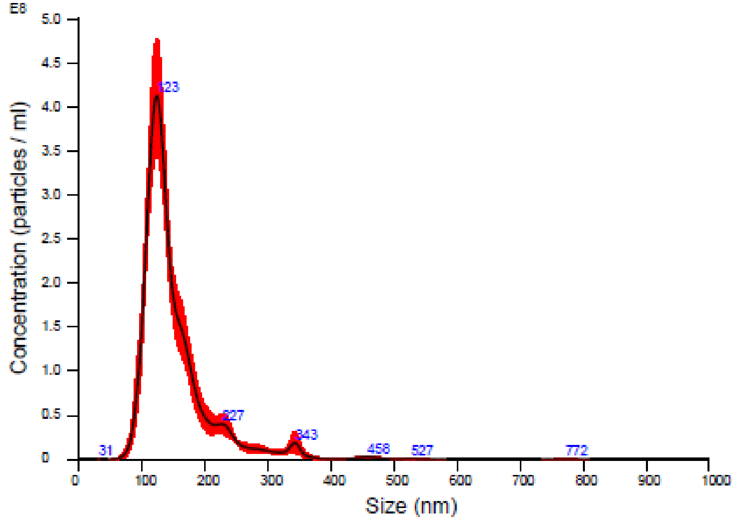

c) MCF10a EVs

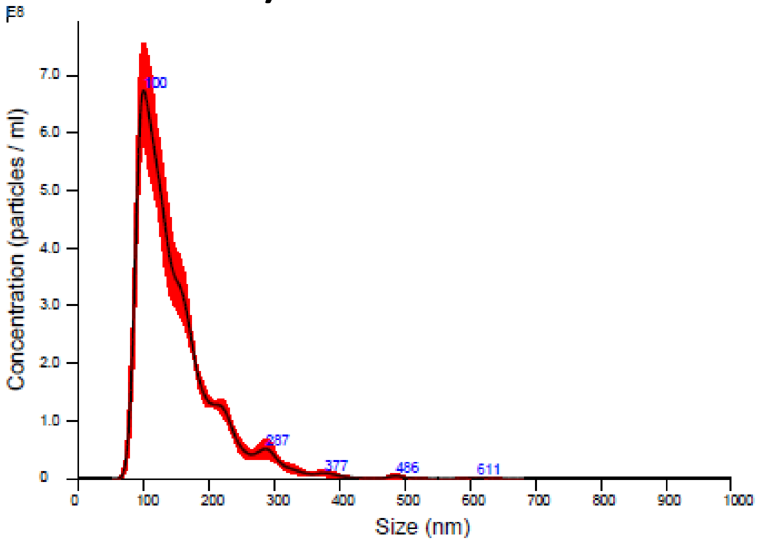

Figure S3. Representative size distribution graphs of EVs observed with the NTA software. Analysis of EVs from a) MDA-MB-231, b) HS578T and c) MCF10a by NS300
